# Editorial: Pediatric Hypertension: Update

**DOI:** 10.3389/fped.2018.00209

**Published:** 2018-07-31

**Authors:** Ibrahim F. Shatat, Tammy M. Brady

**Affiliations:** ^1^Sidra Medicine, Pediatric Nephrology and Hypertension, Doha, Qatar; ^2^Department of Pediatrics, Weill Cornell College of Medicine-Qatar, Ar-Rayyan, Qatar; ^3^College of Nursing, Medical University of South Carolina, Charleston, SC, United States; ^4^Division of Pediatric Nephrology, Johns Hopkins University School of Medicine, Baltimore, MD, United States

**Keywords:** LVH, obesity, microbiome, genetic programming, pheochromocytoma, paraganglioma, developmental origins, kidney transplant

Hypertension in children and adolescents remains a significant health care concern. Epidemiological studies now report that the prevalence of pediatric hypertension ranges from 3% in the general population to up to 25% in obese children. Historically, pediatric hypertension was considered a secondary phenomenon until proven otherwise. However, more recent evidence describes primary hypertension as being more likely than secondary hypertension among children referred to subspecialty care for evaluation of elevated BP in communities where obesity is prevalent. This shift highlights the early relationship between obesity and learned behaviors such as sedentary lifestyle and increased salt/caloric intake with blood pressure.

In this special pediatric hypertension series, we have assembled contributions from global experts in childhood hypertension to provide the reader with a comprehensive and current update on the varied aspects of hypertension diagnosis, secondary etiologies, and cardiovascular co-morbidities. We were fortunate to enlist a prominent group of 22 authors to contribute a wide range of articles. In total, 10 papers have been included (Lewis et al.; Peterson and Miyashita; Al Khodor et al.; Bholah and Bunchman; Woroniecki et al.; Fernandez; Ahn and Gupta; Gurusinghe et al.; Charnaya and Moudgil; Brady).

We introduced the topic of Pediatric Hypertension with a review article highlighting current methodologies and recommendations for hypertension screening in children and adolescents (Lewis et al.). The article by Michaela Lewis, Ibrahim Shatat, and Shannon Phillips walks readers through key issues contributing to both the inaccurate measurement of blood pressure and the misclassification of HTN among children and presents strategies to address these issues. As the authors point out, although national guidelines for the diagnosis and management of pediatric HTN have been available for nearly 40 years, knowledge and recognition of the problem by clinicians remains poor due to a host of influencing factors. They bring to the reader's attention a host of potential exposures known to affect BP, such as recent use of tobacco products, e-cigarettes, consumption of a sodium-rich or high caffeine diet, as well as multiple over-the-counter, herbal, and prescription medications. The authors provided the readers with a comparison between different available measurement devices [Table 1 in (Lewis et al.)]. Last year and after this review (Lewis et al.), the AAP clinical practice guideline for screening and management of high blood pressure in children and adolescents (1) were published. In the new guidelines, the AAP recommends limiting screening BP measurements to preventive care visits for children without risk factors (while continuing to recommend BP screening at all visits for those at increased CVD risk), introduced updated BP tables based solely on normal-weight children, and a simplified BP classification in adolescents ≥13 years of age.

The new guidelines expanded the role for ambulatory BP monitoring (ABPM) in the diagnosis and management of pediatric hypertension. Caitlin Peterson and Yosuke Miyashita contributed an article (Peterson and Miyashita) to this collection emphasizing that 24-h ABPM should be considered standard of care in pediatric patients. In their manuscript, the authors highlight how ABP is superior to clinic BP in evaluating elevated BP and in diagnosis and classification of HTN. In Table 1 of their manuscript, they summarize pediatric studies that examined the association between TOD and ABPM, while in Table 2 they explore potential future clinical applications of 24-h ABPM.

In two of the best written reviews in the field, Sun-Young Ahn and Charu Gupta reviewed for the readers the topic of genetic programming of hypertension (Ahn and Gupta). Table 1 in this review elegantly summarizes monogenic forms of hypertension, while the text discusses methodologies employed in genetic studies of essential HTN, mechanisms for epigenetic modulation of essential HTN, pharmacogenomics and HTN, and recent advances in genetic studies of essential HTN in the pediatric population. Complementing this review, Shari Gurusinghe, Anita Tambay, and Christine Sethna reviewed the developmental origins of hypertension and the role of nephron endowment (Gurusinghe et al.). The authors guide the readers through one of the most intriguing concepts in pediatric nephrology, that is, how the in utero environment may increase the risk of both hypertension and chronic kidney disease. In Figure 2 of their manuscript, the authors propose a flow chart linking low nephron number to hypertension. Furthermore, the authors discuss the impact of ethnicity and postnatal modifiers on nephron numbers.

Tammy Brady contributed a review that focused on the unique aspects of hypertension evaluation and management in the child with comorbid obesity (Brady). Charnaya and Moudgil reviewed the etiology of post-transplant hypertension (Charnaya and Moudgil); they pointed out that HTN is both a risk factor for graft loss and a consequence of multiple transplant related factors including: donor characteristics, recipient factors, medications, and lifestyle attributes similar to those associated with hypertension in the general population. The authors further discuss other useful techniques to assess CVD in this at risk population.

In a comprehensive review of a potentially curable cause of secondary hypertension in pediatric patients, pheochromocytoma (PCC) and paraganglioma (PGL), Bholah and Bunchman point out that these conditions are inherited in as much as 80% of pediatric cases, and that all patients with mutations should be followed closely given the risk of recurrence and malignancy. In figure 1 of their review, the authors reproduced with permission from Lenders et al. (2) a proposed algorithm for genetic testing of patients with PCC or PGL based on clinical characteristics, biochemical phenotype, and tumor pathology. They also outline the pre-, intra- and postoperative management of these challenging tumors as well as follow up.

Woroniecki and colleagues discussed in their review one of the intermediate outcomes resulting from pediatric hypertension: left ventricular hypertrophy (LVH). The review covers the topic from epidemiology to current definitions, clinically relevant methods of left ventricular mass (LVM) measurements (including new methodologies such as cardiac magnetic resonance) and normalization. It also covers clinical management of LVH and how to best achieve regression of LVH in children with HTN (Woroniecki et al.). This review was followed by a commentary by Fernandez in which the author points out that the PESESCAD-HTA study found diastolic abnormalities even in prehypertensive adolescents without LVH, which underlines additional adverse effects of elevated BP and hypertension on the heart.

In the last review of this article collection on pediatric hypertension, Souhaila Al Khodor, Bernd Reichert and Ibrahim Shatat introduce the readers to the relatively new area of investigation in the field of hypertension, posing an interesting question: Can Microbes Regulate Our Blood Pressure? In Figure 2 of their review (Al Khodor et al.), the authors summarize the current hypotheses linking dysbiosis and blood pressure regulation. They point out that while the field is still in its infancy, researchers have started to examine changes in blood pressure when the microbiome is manipulated by dietary and lifestyle changes aiming to achieve a more balanced microbiome.

Our overarching goal of this compilation was to provide the reader with an up-to-date review of pediatric hypertension and to stimulate interest among academic and practicing physicians and scientists on this important topic. With longitudinal studies clearly demonstrating that blood pressure and hypertension in childhood tracks into adulthood, we hope these comprehensive reviews spur more research focused on decreasing the growing burden of hypertension and cardiovascular disease worldwide.

## Author contributions

IS wrote and reviewed the editorial. TB contributed to review process and editing.

### Conflict of interest statement

The authors declare that the research was conducted in the absence of any commercial or financial relationships that could be construed as a potential conflict of interest.

## References

[1] Flynn JT Kaelber DC Baker-Smith CM Blowey D Carroll AE Daniels SR. Clinical Practice Guideline for Screening and Management of High Blood Pressure in Children and Adolescents. Pediatrics (2017) 140:e20171904. 10.1542/peds.2017-1904 28827377

[2] Lenders JW Duh QY Eisenhofer G Gimenez-Roqueplo AP Grebe SK Murad MH. Pheochromocytoma and paraganglioma: an endocrine society clinical practice guideline. J Clin Endocrinol Metab. (2014) 99:1915–42. 10.1210/jc.2014-1498 24893135

